# Evaluation of the morphology of pterygoid hamulus using cone beam computed tomography: A retrospective study

**DOI:** 10.1016/j.jobcr.2025.08.008

**Published:** 2025-08-13

**Authors:** K. Smrithy Sivadas, Vidya Ajila, Shruthi Hegde, Yashika Jain

**Affiliations:** Nitte (Deemed to be University), A B Shetty Memorial Institute of Dental Sciences (ABSMIDS), Department of Oral Medicine and Radiology, Mangalore, India

**Keywords:** Pterygoid hamulus, Forensic, Orofacial pain, Cone beam computed tomography

## Abstract

**Introduction:**

The pterygoid hamulus develops from the medial lamella of the pterygoid process. Understanding the architecture of the pterygoid Hamulus is crucial in terms of image interpretation as well as to diagnose idiopathic pain of the oral cavity and pharynx. Apart from diagnostic implications, the pterygoid hamulus can be utilised in forensic identification by studying its variations in different age groups and genders using three-dimensional imaging modalities such as cone beam computed tomography.

**Materials and methods:**

In this study, 608 Full FOV CBCT images were evaluated for the length, width, inclination, and shape of the pterygoid hamulus on both right and left sides in 4 different age groups, i.e. 20-30, 31–40, 41–50, and 51–60 years and correlated between males and females.

**Results:**

Statistically significant data was obtained with the assessment of pterygoid hamulus length, width, and inclination in the age groups spanning from 20 to 60 years. The distribution of shapes, i.e., slender and triangular, was found to be statistically significant in the assessed age groups. Males had a significantly longer and wider pterygoid hamulus compared to females. No statistically significant data were obtained on mean inclination and shape distribution among males and females.

**Conclusion:**

The assessment of various parameters of pterygoid hamulus using radiographic imaging modalities such as cone beam computed tomography could help in diagnosing orofacial pain of uncertain origin as well as in forensic identification, given variations noticed with age progression and amongst males and females.

## 1 Introduction

Arising from the sphenoid bone's medial pterygoid plate base, the pterygoid hamulus (PH) is a curved, narrow bony projection that extends downward and laterally. PH supports various tissues such as tensor veli palatini muscle, pterygomandibular ligament, superior half of the pterygopharyngeal sphincter muscle, and buccinator muscle.^1^^,^^2^

The PH rests at the lowermost end of the posterior border of the medial pterygoid plate curling outward with a groove for the tendon of the tensor veli palatini muscle. The muscle fibres go down to the perimeter of the medial pterygoid plate where a steep right-angle curve is made around the hamulus before it extends horizontally toward the centre to form the soft palate's palatine aponeurosis. Furthermore, the pterygoid hamulus serves as an anchor point for the pterygomandibular raphe, a structure that connects the buccinator muscle to the superior constrictor muscle of the pharynx. The bursa located between the neck groove of PH and the muscular tendon guarantees the fineness in soft palate movement. The superior half of the pharyngeal sphincter regulates the air pressure in the middle ear during various actions like the sucking, swallowing, chewing, sneezing and yawning; therefore, maintaining airway patency during sleep.^3^

Elongation syndrome is the most prevalent condition linked with PH, with a prevalence rate of 1 %. Elongation of PH is characterized by various manifestations of the palatal and pharyngeal regions, including pain as well as discomfort during swallowing. Hjort-Hansen et al. coined the name "pterygoid hamulus syndrome" [PHS] to characterize pain in the palate and pharyngeal regions caused by morphologically altered PH, with approximately 40 cases reported in the literature since 1987.^4^

Numerous investigations on the location and shape of PH in various populations have been carried out, and based on those investigations, the length of PH was determined to be between 4.9 and 7.2 mm.^5^ The sagittal and transverse dimensions of PH were similarly reported by Putz and Kroyer^2^ to be 1.4 mm and 2.3 mm, respectively, while Sasaki et al.^6^ also reported an extended PH length of 13 mm.

According to Krmpotic-Nemanic et al.^3^ and Orhan et al.,^7^ the PH showcases atrophy with advancing age, leading to constriction of the upper pharynx as a result of lack of muscle support. This structural insufficiency can cause sleep apnea and disordered breathing.

Apart from these, the variations of PH morphology can be used in the field of forensic odontology for the identification of the deceased. The present study was undertaken to assess and compare PH parameters in different age groups as well as between males and females using cone beam computed tomography [CBCT], a 3D radiographic imaging modality.

## 2 Materials and Methods

### 2.1 Study design

This retrospective radiographic study was conducted in the Department of Oral Medicine and Radiology**.** Full Field of view [FOV] CBCT images obtained using the CBCT unit (Planmeca Promax 3D Mid, Helsinki, Finland) between January 2021 to January 2024 were utilised for the study. Ethical clearance [ETHICS/ABSMIDS/498/2024] for the study was obtained from the institutional ethical committee.

All included scan volumes had been obtained using the same CBCT unit with standard imaging protocols, under 16 × 10.2 cm field of view (FOV); voxel size 0.4 mm and exposure settings were standardised at 90 kVp, 10 mA and 8–13 s. The Full FOV scans which met the inclusion criteria were segregated from the department database and were not specifically acquired for this study. The scans were of patients referred for radiographic evaluation as part of orthodontic treatment; facial bone evaluation following trauma and those with temporomandibular joint disorders.

### 2.2 Sample calculation

Based on Pooled standard deviation of length of PH in males 2.705 and in females 2.665,^13^ mean difference 1, effect size 0.3724, α error 5 %, power 90 % for two-sided test, sample required per group was 152. This was calculated using nMaster software version 2. The sample size was calculated as follows:

n = 2s p ^2^[Z _1-α/2_ + Z _1-β_] ^2^ / μ^2^_d_; s^2^_p_ = s_1_^2^ +s^2^_2_ /2

where s_1_^2^ means the standard deviation in the first group, s^2^_2_ means the standard deviation in the second group, μ^2^_d_ means mean difference between the samples, α being the significance level and 1-β being the power.

### 2.3 Study population and inclusion criteria

The study population predominantly consisted of South Indian patients hailing from the states of Karnataka and North Kerala, India. CBCT volumes of subjects within the age range of 20–60 years and with a full complement of teeth in both the maxillary and mandibular arches met the inclusion criteria. The CBCT volumes were categorised into 4 groups with age ranging from 20 to 30, 31–40, 41–50 and 51–60 years respectively. Patients with a history of mandibular fractures, severe developmental anomalies like micro- or macrognathia, temporomandibular joint [TMJ] ankylosis, and any bony lesion were excluded from the study.

Based on the inclusion criteria, 152 scans were enrolled in each group, for a total assessment of 608 CBCT scans, with similar distribution among male and female subjects in each age group.

### 2.4 Measurement of parameters

The CBCT images were analysed using Romexis software version 4.6.2.R. The PH parameters assessed were the length, width, inclination, and shape bilaterally.

**Fig. 1:**
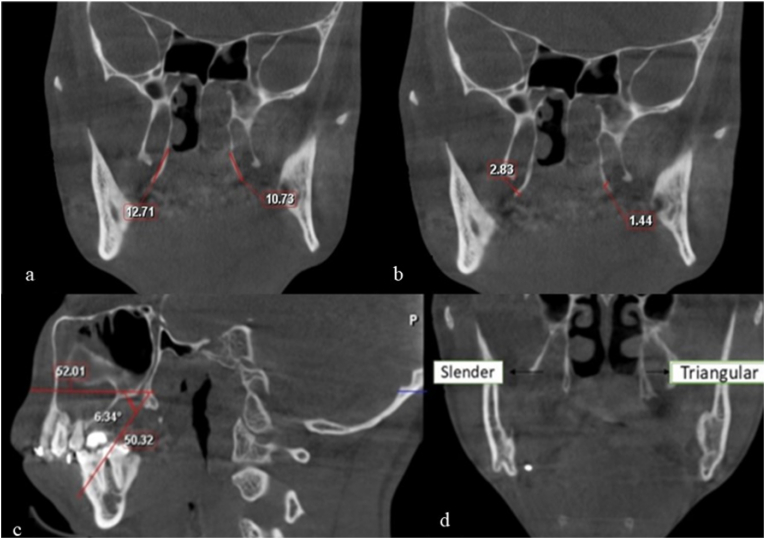
[a] PH length measured from base of medial pterygoid to tip of PH. [b]: PH width measured at the most prominent part of the PH. [c] PH inclination measured on the sagittal section. [d] Shape of PH assessed as slender and triangular.
b] *PH width*: Measured at the most prominent part of the PH bilaterally [Fig. 1b].
c] *PH shape*: Categorised as Slender or Triangular by assessing its morphology bilaterally [Fig. 1c].

The coronal CBCT section where both the right and left PH were visible was identified and utilised for the assessment of PH parameters. The parameters were assessed with the methodology adapted from the investigation carried out by Oz et al.^8^ The parameters assessed were.

a] *PH length*: Measured from the base of the medial pterygoid to the tip of the PH [Fig. 1a].

The sagittal section, where the anterior nasal spine and PH were visible in a single section was utilised for the assessment of the PH inclination.

d] *PH inclination*: Two lines were drawn; one along the anterior nasal spine to the base of the PH and from the base of PH to the tip. The angle formed between these lines was measured to obtain the inclination [Fig. 1d].

The radiologist who assessed the scans was blinded about the clinical diagnosis, age and gender of the patient. Measurements were done using the measurement tools of the Romexis imaging software. The measurements were made by a single radiologist and repeated after an interval of 2 weeks. The average of the measurements was taken as the final value.

The data obtained was entered into the excel sheet and was subjected to statistical analysis.

### 2.5 Statistical analysis

The data was analysed using Statistical Package for Social Sciences [SPSS] software for Windows [ver. 26.0, IBM Corp., Armonk, NY]. Continuous data between the groups was analysed using One-way analysis of variance [ANOVA], followed by a post-hoc test. Categorical data were compared between the groups using chi-square test. Results were presented using graphs and tables. The level of significance was set at P < 0.05. With a 5 % alpha error, the study presented a power of 90 %.

## 3 Results

**Fig. 2:**
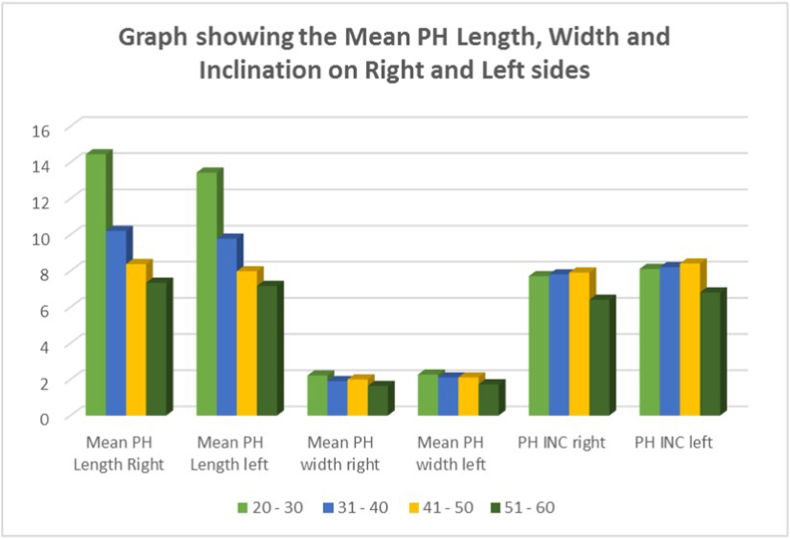
Graph showing the Mean PH Length, Width, and Inclination on the Right and Left sides. The post hoc multiple comparison Tukey's test between groups revealed significantly higher PH length, width, and inclination in the age group of 20–30 years, with the 51–60 years' age group having the smallest PH width and inclination.

The assessment of length of PH with respect to the right as well as the left side showed a maximum length in the age group of 20–30 years [14.44 mm and 13.43 mm] with a gradual decline in the 51–60 years age group [7.34 mm and 7.16 mm]. There was a statistically significant difference in the PH width between the age groups, with the width being maximum in the age group of 20–30 years [2.21 mm and 2.26 mm]. The mean difference in inclination of PH was statistically significant (P = 0.001) between different age groups in relation to both right as well as left sides, with the age group of 41–50 years exhibiting the maximum inclination (7.7° and 8.1° respectively). The data distribution is given in Fig. 2.

**Fig. 3:**
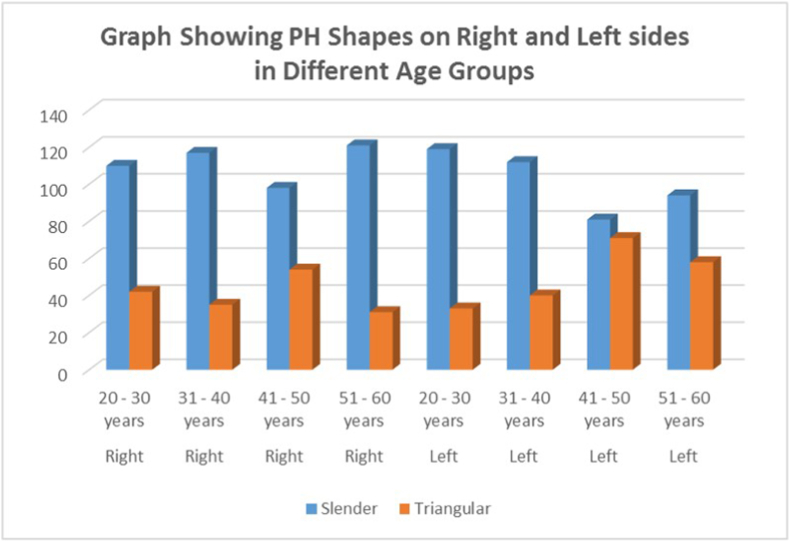
Graph showing shapes of PH on the Right and Left sides in different age groups.

On comparison of the PH shape with respect to right and left sides, it was found that in the age group of 41–50 years, 33.3 % and 35.1 % of participants respectively had triangular-shaped PH while the slender shape was exhibited by 22 % and 20 % on right and left sides respectively (Fig. 3). The distribution was statistically significant (P = 0.001) for both sides.

Males had a statistically longer and wider PH compared to females with respect to both right and left sides. There existed no statistically significant difference in the mean inclination of PH between males and females on both the right (P = 0.61) as well as the left side (P = 0.86). Though males exhibited more of triangular-shaped PH than females who had slender-shaped PH, the distribution was not statistically significant [Table 1].

**Table 1:** Comparison of mean PH length, width, and inclination and shape between males and females.

			Mean	SD	t	P value
PH Length (mm)	Right Side	Males	10.44	3.79	2.3	P = 0.02∗
		Females	9.72	3.8		
	Left Side	Males	9.8	3.5	1.9	P = 0.05∗
		Females	9.3	3.3		
PH Width (mm)	Right Side	Males	2.02	0.72	2.9	P = 0.003∗∗
		Females	1.86	0.63		
	Left Side	Males	2.07	0.79	0.76	P = 0.44
		Females	2.03	0.78		NS
PH INC (in degrees)	Right side	Males	7.3	3.5	−0.5	P = 0.61
		Females	7.55	3.9		NS
	Left Side	Males	7.8	3.7	−0.16	P = 0.86
		Females	7.9	3.8		NS

Shapes		*Slender*	*Triangular*	*P value*
		N (%)	N (%)	
Right Side	Males	219 (49.1)	90 (55.6)	P = 0.17
	Females	227 (50.9)	72 (44.4)	NS
	Total	446	162	
Left Side	Males	200 (49.3)	109 (54)	P = 0.3
	Females	206 (50.7)	93 (46)	NS
	*Total*	*406*	*202*	

SD−standard deviation; PH-Pterygoid Hamulus; INC-inclination; mm-millimetres.

NS-not significant and statistically significant at ∗P < 0.05 &∗∗P < 0.01 using unpaired *t*-test.

The intra examiner reliability was assessed for the radiographic interpretations and was found out to be significant for the length, width and inclination of PH [Table 2].

**Table 2:** Intra-examiner reliability for radiographic interpretations.

		ICC	95 % CI	P value
Length	Right side	0.98	0.97–0.99	P = 0.001∗
	Left side	0.98	0.97–0.99	P = 0.001∗
Width	Right side	0.76	0.7–0.82	P = 0.001∗
	Left side	0.85	0.78–0.89	P = 0.001∗
Degrees	Right side	0.97	0.96–0.98	P = 0.001∗
	Left side	0.99	0.99–0.99	P = 0.001∗

ICC- Inter class correlation coefficient; CI - Confidence Interval.

## 4 Discussion

The term "pterygoid," which means shape like a wing, has origins from the Greek words ‘pteryx’ (wing) and ‘eidos’ (-like). It thus refers to an osseous segment that extends from the lowermost portion of the sphenoid bone's medial pterygoid plate and is constituted by base, body, head, and neck. PH morphology changes as a result of the position, length, and inclination of muscles like the pars pterygopharyngea, palatopharyngeus constrictors, and tensor veli palatini being subjected to unbalanced stresses.^9^

Numerous investigations have assessed PH morphology with or without clinical correlations using cadaveric, radiographic, and combination techniques. The knowledge regarding PH morphology facilitates the diagnosis of orofacial pain without any contributing etiological variables.^10^ In addition, understanding the morphological changes in PH between males and females, and in different age groups, opens up new avenues in forensic odontology to identify the deceased individuals. Thus, this study was conducted to analyze PH parameters based on age and gender using a 3D radiographic modality like CBCT.

In our study, the PH length on both right and left sides gradually declined with age advancement, with the shortest length in the age range of 51–60 years. The increase in PH length between age groups was significant, with the 20–30 years’ age group showcasing the maximum PH length, with a gradual decline in the age range of 51–60 years. The findings of this study were compatible with those of Krmpotic-Nemanic et al.^3^ who stated that children had the shortest PH, which increased in adults and reduced dramatically in the elderly. The short PH length present during infancy gradually elongates, with PH extending up to the upper section of the buccopharyngeal raphe, in turn getting attached to the jaw bones. Tooth loss generates biomechanical pressure on PH, resulting in shortening in old age.^11^ In the present study, we noted a decrease in PH length with advancing age, even though the subjects had a full complement of maxillary and mandibular teeth. The variation in the length of the PH noted as age advances may have consequences for PHS. PHS results in pain and discomfort in the pharyngeal and palate areas and can be linked to elongated hamuli, particularly those with an incorrect inclination.^12^ In the study by Oz et al., the authors found an inverse relationship between the length of PH and apnea-hypopnea index, with a higher incidence of Obstructive sleep apnea (OSA) in the elderly population with shorter PH length. The spatial features of PH influence the activity of muscles like tensor veli palatini which prevent the collapse of the upper airway.^8^

The data from this study showed the PH length to be significantly longer in males than in females, with the largest dimensions being 10.44 ± 3.79 mm and 9.80 ± 3.5 mm, respectively. These findings are consistent with the data obtained from studies conducted by Romoozi et al.,^12^ Mehra et al.,^13^ and Nerkar et al.,^14^ who postulated that males had significantly longer average PH lengths than females. As PHS is thought to have a male preponderance based on the reported cases in the literature, the increased length of PH noted in males might predispose them to PHS, hence enabling early diagnosis and management of the same. The variation in PH length noted between males and females may also have implications for gender determination in forensic sciences.^15^

The PH width was significantly higher in males than in females on both right and left sides, which is in accordance with the study conducted by Nerkar et al.^14^ and Mehra et al.^13^ The width of PH showed a statistically significant decline from the age range of 20–30 years to 51–60 years with respect to both sides. These findings were in line with the data of Orhan et al.,^8^ who demonstrated the width of PH decreasing from 1.85 mm to 1.73 mm as age shifted from the younger to the older age group, and with that of Romoozi et al.^12^ who discovered that the average PH width on both sides reduced with aging. Although there is uncertainty regarding the theory behind the disparity in width between the left and right PH, it is possible that the variations in the manner in which stomatognathic muscles distribute their stresses could be a factor in the PH's altered dimensions.^16^ In the present study, we found a statistically significant difference in the width of PH on the right and left sides.

The posterior inclination of the PH measured in the sagittal plane showed a statistically significant reduction with age on both sides. The values ranged from 7.7 ± 3.8° and 8.1 ± 3.8° in the age group of 20–30 years to 6.4 ± 3.1° and 6.8 ± 3.2° in the age range of 51–60 years with respect to both right and left sides. Romoozi et al.^12^ discovered a similar finding, which showcased that as age advances, the PH slope in the sagittal plane decreases on both sides due to uneven stresses and reduced bone density. The present study revealed no statistically significant difference in the mean inclination of PH between males and females on both the right as well as left sides, which is in accordance with the investigation carried out by Khoubivand et al.^17^ The variation in the inclination of PH might contribute as an etiological factor for orofacial pain of idiopathic origin, as it might irritate the surrounding tissue and muscles, igniting pain and discomfort in the patient.^18^

The distribution of shapes of PH, i.e., triangular and slender with respect to right and left sides, showed triangular shape being more prevalent than slender form in the various age groups, which is in accordance with the study by Nerkar et al.^14^ The study by Song et al.^19^ found the slender form to be more prevalent than the triangular form, which contradicted the findings of the present study. Although males had more triangular-shaped PH and females had slender-shaped PH, there was no statistically significant difference in PH shape between the genders. While the two morphologically recognized forms of PH are triangular and slender, any variation may irritate the soft palate and pharyngeal regions, causing pain to be referred to the palate, pharynx, or even temporal area, mimicking other conditions such as temporomandibular joint disorders (TMDs) or trigeminal neuralgia. Thus, abnormal PH shape may be a source of orofacial pain of unknown origin.^20^

It is imperative to note from a clinical perspective that the PH is undoubtedly a possible source of irritation due to its tendon attachments and the "pulley" it offers to the muscle tensor veli palatini. A morphological anomaly of PH may cause discomfort by impairing the tensor veli palatini muscle's contraction, causing the tensor veli palatini bursa to be inflamed or fibrosed as a result of increased pressure on the palatine aponeurosis, or by mechanically irritating the surrounding tissues.^21^ This has the potential to stimulate nerves like greater palatine, lesser palatine, facial, or glossopharyngeal nerves, which could result in pain in different parts of the maxillofacial region. The main diagnostic conundrum regarding the diagnosis of patients with elongated PH is that it is not associated with any specific clinical manifestations.^22^

Ethnic differences between populations might also contribute to the variations noted in PH parameters. Variations in genetics, skeletal development, and functional adaptations are the main causes of ethnic disparities in PH characteristics. The hamulus's size, shape, and location are eventually shaped by these elements, which also affect bone growth and remodelling. Various populations may exhibit diversity in hamulus morphologies due to variations in specific genes and proteins involved in bone formation and development. The pterygoid hamulus grows and changes throughout life due to various factors, including mechanical pressures, nutrition, and heredity. The timing and degree of hamulus growth can vary according to the ethnic group's skeletal development.^23^ Two schools of thought are accepted in the view of influence of ethnicity on the morphology of PH. While one school of thought expresses that PH variations are based mainly on sex rather than ethnicity, other authors have investigated the PH shape to be more of triangular form in Indian population compared to slender form being common amongst Iranian population.^11^ Hence, ethnical influence may have a role in PH morphology apart from the variations due to age and gender.

The findings from this study suggest that accurate identification of unexplained pain in the oropharyngeal area requires considering anatomical variations in PH dimensions with age and gender. Diagnosing the signs and symptoms of these alterations might be challenging without considering the underlying anatomy. In addition, the above data regarding the morphological variations of PH noticed with age and gender can be utilised as a supplementary tool for gender identification in forensic sciences. The variations of PH studied with gender and advancing age can be incorporated for forensic age determination, with this study being the first of its kind to evaluate age and gender changes in PH, thereby paving the way for a new arena for future research.

## 5 Clinical significance of the study

The primary outcome of the study was that PH showed significant variations between males and females and with advancing age. This has implications in forensic science as it can be used as a supplementary tool for identification of the deceased. Although skeletal characteristics and tooth measurements are the mainstay approaches utilised in the forensic field for sex determination and age assessment, the assessment of pterygoid hamulus can be incorporated as a potential supplementary tool, particularly when typical anatomical markers are unavailable, as in intricate situations involving incomplete or fractured skeletal remains for identification of the deceased. Further, variations of PH could be an additional factor in patients of various ages presenting with orofacial pain of unknown origin, which will help in clinical diagnosis.

The secondary outcome obtained from the study is that the variation of length exhibited by PH when combined with the apnea-hypopnea index may identify the population susceptible to contract OSA. Apart from the standardised shapes of PH recognized in literature, i.e., slender and triangular, any morphological alterations may irritate the associated musculature, leading to pain in the oropharyngeal region. The variation in the inclination of the PH may also cause irritation to the musculature resulting in altered sensation in the orofacial region.

## 6 Limitations of the study

Since the study was retrospective, and data from the department database was analysed, the clinical assessment of the study population was limited to the data in the CBCT volumes and patient case sheet. Due to the absence of clinical data in the present study, the exact relationship of the PH morphology with definite clinical signs and symptoms could not be carried out. As the study followed a restrospective pattern,the dynamics exhibited by PH as an interplay of its functional and anatomical components on the stomatognathic system and the subsequent clinical presentations that prevail, could not be studied on a broader aspect. Future studies could adopt a prospective design with a larger sample size by combining the clinical evaluation of the subjects along with radiological assessment of PH parameters. Due to the nature of the present study, soft tissue correlation could not be done as soft tissue details are not clearly visualised in CBCTs.

## 7 Future perspectives

Apart from the three dimensional imaging modality utilised in this study, a multimodal imaging strategy that combines the advantages of imaging modalities such as MRI, CT, ultrasound, and optical imaging techniques can be used to efficiently investigate soft tissue interactions surrounding the pterygoid hamulus. A thorough evaluation of the pterygoid hamulus is possible by combining the soft tissue resolution of MRI with the bony window of CT. The real-time imaging capabilities of ultrasound can offer a dynamic perspective of the region and the tissues around it. To gain a better understanding of the function, optical imaging can be used in conjunction with other modalities to observe cellular processes or structures within the soft tissues. Also, the use of automated tools for measurement of parameters can enhance data reliability apart from the software measurement tools.

## 8 Conclusion

Understanding the architecture of anatomical structures aids in imaging interpretation and offers important information regarding the differential diagnosis of untraceable pain pertaining to the pharynx and oral cavity. Evaluation of PH morphology is essential in patients with vague orofacial pain as it may be related to PH elongation and inclination. Further research should focus on exploring the potential of using PH measurements as a supplementary tool in the assessment of forensic age and gender of the deceased. However, due to the retrospective nature of the present study, the correlation between the functional and morphological aspects of PH with the clinical presentations of the patients could not be assessed. The influence of PH on its surrounding soft tissue structures were not assessed due to the limited soft tissue resolution of CBCT. The ethnical diversity of PH should not be overlooked and future studies should invest in understating the diversity manifested by PH within and between various ethnical populations.

## Patient's/Guardian's consent form

Waiver of informed consent form as data was retrieved from the department database.

## Ethical clearance

Institutional Ethical Clearance obtained for the study [ETHICS/ABSMIDS/498/2024].

## Sources of funding

The research article has obtained no funding from any sources.

## Declaration of competing interest

The authors declare that they have no known competing financial interests or personal relationships that could have appeared to influence the work reported in this paper.

## Abbreviations

PH: Pterygoid Hamulus
PHS: Pterygoid Hamulus Syndrome
CBCT: Cone Beam Computed Tomography
OSA: Obstructive Sleep Apnea
FOC: Field of View
TMJ: Temporomandibular Joint
SPSS: Statistical Package of Social Sciences
ANOVA: Analysis of variance
TMDs: Temporomandibular joint disorders
